# Need-based remote exercise management platform for colorectal cancer patients with intestinal stoma: design and pilot usability evaluation

**DOI:** 10.3389/fresc.2025.1633231

**Published:** 2025-08-06

**Authors:** Cui Yao, Lingyu Ding, Jing Yang, Yueming Sun

**Affiliations:** ^1^ Department of General Surgery, The First Affiliated Hospital with Nanjing Medical University, Jiangsu, Nanjing, China; ^2^ The First School of Clinical Medicine, Nanjing Medical University, Nanjing, China

**Keywords:** colorectal cancer, intestinal stoma, exercise, remote platforms, SUS

## Abstract

**Objective:**

To develop and evaluate the usability of a comprehensive remote management platform specifically designed to promote exercise among colorectal cancer patients with intestinal stomas.

**Methods:**

We employed a multifaceted approach to platform development, integrating systematic literature reviews, semi-structured patient interviews, healthcare provider consultations, and interdisciplinary expert panel discussions to identify the unique exercise management needs of colorectal cancer patients with intestinal stomas. Based on these comprehensive needs assessments, we constructed a specialized remote exercise management platform. Platform usability was subsequently evaluated using the validated System Usability Scale (SUS).

**Results:**

The finalized platform architecture comprised two interconnected interfaces: a patient-centered portal and a healthcare provider administrative system. Usability evaluation was completed by 62 colorectal cancer patients with intestinal stomas, yielding a mean SUS score of 88.39 ± 3.65, substantially exceeding benchmark standards for digital healthcare applications.

**Conclusion:**

The developed platform demonstrates exceptional usability while offering comprehensive functionality, evidence-based content with rigorous medical validation, and robust security features. This technological solution provides a practical framework for enhancing exercise management quality in colorectal cancer patients with intestinal stomas and establishes a blueprint for similar applications addressing postoperative rehabilitation needs in other clinical populations.

## 1 Introduction

As a top-five cancer in China by incidence and mortality, colorectal cancer remains a critical public health challenge, severely impacting population health and longevity (1, 2). Intestinal stoma creation remains a standard surgical intervention in colorectal cancer management (3). During postoperative rehabilitation, the presence of an intestinal stoma imposes considerable adverse effects on patients across multiple domains. From a physiological perspective, stoma management presents complex challenges including the necessity for frequent appliance changes, which may precipitate complications such as peristomal dermatitis and stomal infection. Furthermore, patients face risk of serious complications including parastomal hernia, stomal stenosis, and stomal hemorrhage, all of which significantly compromise quality of life (4–6). From a psychological standpoint, the alteration in body image consequent to stoma formation frequently engenders feelings of diminished self-worth, heightened anxiety, and clinical depression (4–6). Consequently, optimizing rehabilitation protocols and enhancing care quality for this vulnerable patient population constitutes a clinical imperative of paramount importance (7).

Exercise constitutes a fundamental rehabilitation component for colorectal cancer patients with intestinal stomas (8). The American Cancer Society's guidelines for colorectal cancer survivorship care explicitly recommend regular moderate-intensity physical activity for all colorectal cancer survivors (9). Substantial evidence demonstrates that structured, evidence-based exercise interventions significantly improve survival outcomes, physical functioning, and psychological well-being among colorectal cancer patients (10–12). Furthermore, targeted core muscle strengthening regimens effectively reduce the incidence of common stoma-related complications, particularly parastomal hernia formation (13). Despite these documented benefits, the majority of colorectal cancer patients with intestinal stomas fail to achieve guideline-recommended physical activity levels, with many avoiding exercise entirely due to apprehension regarding potential adverse effects from improper movement patterns (14). Consequently, facilitating appropriate exercise adoption during post-discharge rehabilitation represents a critical clinical objective. Remote management platforms have emerged as essential components of extended care services, gaining widespread implementation in chronic disease management. These technological solutions significantly enhance behavioral adherence through their accessibility, real-time monitoring capabilities, and efficient data management functionalities (15–17). Previous studies have shown that digital health platforms play a crucial role in supporting exercise for ostomy patients. They provided exercise guidance and remote monitoring to help patients safely and effectively engage in rehabilitation training (18, 19). Nevertheless, the design of prior telemanagement platforms frequently overlooked the key needs of patients, resulting in a constrained impact on their clinical implementation (20).

In summary, we aim to develop a remote management platform focusing on exercise management for colorectal cancer patients with intestinal stoma from the perspective of patient needs and evaluate the usability of this platform.

## 2 Methods

**Figure 1:**
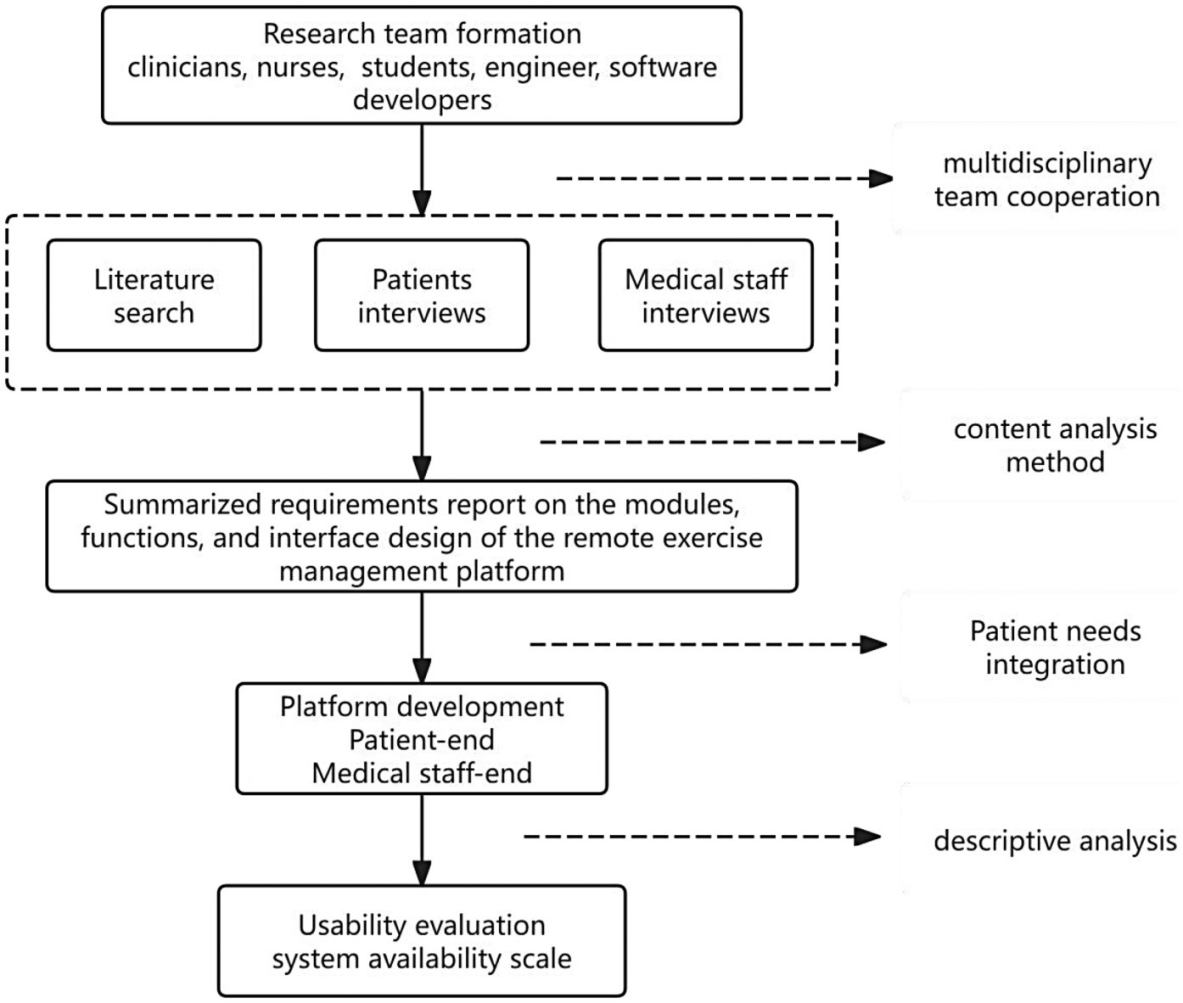
The whole process of this study.

The whole process of this study is shown in Figure 1.

### 2.1 Development process of remote exercise management platform

#### 2.1.1 Research team formation

The multidisciplinary team consisted of 3 clinicians and 7 nurses (medical support and follow-up), 2 postgraduate students (literature review and data analysis), and 3 technical members (platform development, testing, and maintenance).

#### 2.1.2 Literature review

Literature search was used to obtain the remote exercise needs of patients with colorectal cancer stoma. PubMed (Medline), Embase, Web of Science, The Cochrane Library, CINAHL, Proquest, China National Knowledge Infrastructure (CNKI), and Wanfang databases were retrieved. The search terms included MeSH terms and free text words, including rectum cancer, colon cancer, surgery, intestinal stoma, exercise, demand, and remote platforms.

#### 2.1.3 Patients needs and requirements assmessment

To obtain more targeted results, we interviewed patients and medical staff to obtain their needs and requirements. The interviewers had rich interview experience and received professional training.

##### 2.1.3.1 Patients interviews

We used face-to-face interviews to obtain patients' requirements for the design of a remote exercise management platform. Inclusion criteria for patients: diagnosed with colorectal cancer and underwent ostomy surgery; age ≥ 18 years old; awareness of disease diagnosis own disease diagnosis; volunteer to participate in this interview and sign informed consent. The outline of the interview was as follows: What are your concerns and difficulties with regular exercise after discharge from the hospital; what kind of help do you need from the medical staff when you conduct regular exercise; how do you think about the remote management platform to help you with exercise management after discharge from the hospital; what functions would you like to receive in this remote management platform; do you have any other suggestions for the content design of this remote management platform. According to the interview outline, 20 colorectal cancer patients with intestinal stoma were selected to be interviewed for 20–40 min until information saturation. The interview location was the departmental demonstration classroom, and the environment was kept quiet during the interview to protect the patients' privacy fully.

##### 2.1.3.2 Medical staff interviews

Face-to-face focus group interviews were conducted to obtain medical staff's suggestions and needs for the main modules and functions of the remote exercise management platform. Inclusion criteria for medical staff: with 5 years or more clinical work experience in colorectal cancer-related fields; have 2 years or more clinical or scientific research experience in the field of exercise management; volunteer to participate in this interview and sign informed consent. Interviews were conducted with 10 clinicians and nurses in the colorectal cancer specialty for over 5 years. The interviews centered on the following topics, which were discussed in depth until no new ideas emerged: What do you think are the difficulties in regular exercise after discharge for colorectal cancer patients with intestinal stoma; how do you think the remote exercise management platform should help patients better achieve regular exercise after discharge; What are your thoughts on the module setting and function realization of the remote exercise management platform; what are your needs and suggestions for the use of the medical and nursing side of the remote exercise management platform.

##### 2.1.3.3 Analysis of interview text

This study used a content analysis method for analysis, with specific steps as follows: determine the sentences related to patients' requirements for the design of the platform and medical staffs' needs for the main modules and functions of the platform as the smallest analysis unit; read the original materials multiple times, and carefully study the textual content; classify the extracted requirements as the main theme coding names; explain and illustrate the results, establish the relationship between the text content and the extracted main themes and sub-themes.

#### 2.1.4 Patient needs integration

By integrating the results of the literature review, patient interviews, and medical staff interviews, we summarized the requirements report on the modules, functions, and interface design of the remote exercise management platform. Through the expert group discussion method, 10 clinical and nursing experts from colorectal specialties were invited to discuss the requirements requirements report further. The expert discussions focused on several key aspects: Assessing the comprehensiveness of the module settings of the remote exercise management platform and determining whether additions, deletions, or modifications are necessary; evaluating the rationality of the functional implementation of the platform and identifying any required adjustments; examining whether the operational mechanisms of the remote exercise management platform can effectively meet the exercise support needs of patients and deciding if any modifications are needed.

#### 2.1.5 Platform development

The final design needs of the module setting and function realization of the remote exercise management platform were handed over to the software developers to form the first version of the remote exercise management platform. Ten patients and five medical staff were recruited to test the functionality of the initial version of the management platform. At the end of the test, the researcher conducted focus group interviews with 15 testers to obtain respondents' experiences during the testing process. The software developers further improved the modules and functions of the remote exercise management platform according to the revised content from the group interviews.

### 2.2 Usability evaluation of remote exercise management platform

#### 2.2.1 Participants

The convenience sampling method was used to select colorectal cancer patients with intestinal stoma in the colorectal surgery department of a hospital in Jiangsu Province from April to June 2023. Inclusion criteria: diagnosis of colorectal cancer confirmed by colonoscopy and pathological examination; intestinal intestinal stoma surgery to be performed; age ≥ 18 years. Exclusion criteria: having a severe physical disability or cognitive impairment, a combination of tumors from other sites. Studies have shown that using the System Usability Scale (SUS) to collect small sample data of 12–14 people can achieve 90% to 100% accuracy (21).

#### 2.2.2 Usability testing

##### 2.2.2.1 General information questionnaire

It was compiled by the researchers themselves after clinical research and review of relevant literature and mainly included general demographic information such as age, gender, and education level.

##### 2.2.2.2 System usability scale

The System Usability Scale (SUS) (22) was used to evaluate the usability of the remote exercise management platform. The SUS scale is a 10-item questionnaire scored on a 5-point likert-type scale from 1 (strongly disagree) to 5 (strongly agree). Its advantages include versatility, simplicity, low cost, accuracy, and validity. Its reliability (Cronbach *α*=.85) has been reported (22). The questionnaire is designed to be answered after the user's interaction with the system. It is arranged to alternate between positive and negative statements to avoid habitual bias from the respondent. The score contribution for the odd items (the positive statements) is the scale position minus 1 and the contribution for the even items (the negative statements) is 5 minus the scale position. The overall score is calculated from the sum of all item scores multiplied by 2.5, with the overall score ranging from 0 to 100. A system with a score above 85 is considered to have excellent usability, whereas a system with a score between 68 and 84 is considered to have good usability. In this study, patients were asked to complete the SUS after four weeks of continuous use of the remote exercise platform after discharge. The items in SUS are shown in Appendix 1.

#### 2.2.3 Descriptive statistics

The approach to characterizing data varied based on the type of data and its distribution. When dealing with measurement data that adheres to a normal distribution, the mean and standard deviation were employed to offer a clear and concise depiction. Conversely, for measurement data that deviates from a normal distribution, the median and interquartile range served as the preferred metrics for an accurate portrayal. As for count data, frequency and percentage were utilized to provide a straightforward and informative summary.

## 3 Results

### 3.1 Interview results

A total of 10 patients and 10 medical staff were included in this study for interviews. The general information of the interviewees is shown in Table 1. In the patient interview, through the analysis of the interview text, a total of 2 themes and 6 sub-themes were extracted. In the interview of medical staff, a total of 3 themes and 6 themes were extracted, as shown in Tables 2, 3.

**Table 1:** General information of the interviewees.

Patients					Medical staff				
No.	Gender	Age(years)	Education level	Career	No.	Gender	Age(years)	Education level	Career
P1	Female	60	Junior school	Farmer	N1	Female	30	Bachelor degree	Nurse
P2	Male	53	Bachelor degree	Teacher	N2	Female	45	Master degree	Nurse
P3	Male	56	Junior school	Security personnel	N3	Male	41	Master degree	Doctor
P4	Male	68	Junior school	Farmer	N4	Male	39	Doctoral degree	Doctor
P5	Female	68	Senior school	Office worker	N5	Female	30	Doctoral degree	Nurse
P6	Male	45	Bachelor degree	Exposed workers	N6	Female	41	Bachelor degree	Nurse
P7	Male	61	Senior school	Exposed workers	N7	Male	46	Doctoral degree	Doctor
P8	Male	61	Senior school	Office worker	N8	Male	55	Master degree	Doctor
P9	Male	41	Bachelor degree	Exposed workers	N9	Female	28	Bachelor degree	Nurse
P10	Female	69	Junior school	Office worker	N10	Female	39	Bachelor degree	Nurse

**Table 2:** The results of patients interviews.

Themes	Sub-themes	Description of connotation
Exercise content	Personalized exercise program	Patients hope to develop an exclusive exercise program based on their physical condition, stoma type, exercise ability and other factors.
	Exercise safety guidance	Patients want to know which movements need special attention, and how to correctly wear auxiliary equipment such as pockets to ensure safety during exercise.
	Exercise difficulty classification	Patients hope to gradually achieve exercise progression according to their own recovery, from simple rehabilitation exercise to a certain intensity of enhanced physical exercise.
Platform function	Professional medical support	Patients expect the platform to have professional medical staff to provide advice and guidance online, so that they can get professional answers in time when they encounter problems during the exercise.
	Motion data monitoring and feedback	Patients expect the platform to have motion data monitoring functions, such as recording exercise time, exercise intensity, heart rate, etc., and provide patients with personalized motion feedback and suggestions based on these data to help them better adjust their exercise plans.
	Social interaction function	Patients expect the platform to have a social interaction section, so that they can communicate with other stoma patients about sports experience and encourage each other.

**Table 3:** The results of medical staff interviews.

Themes	Sub-themes	Description of connotation
Patient management and monitoring	Patient data tracking	Medical staff hope that the platform can track the patient's motion data in real time, so as to keep abreast of the patient's movement and physical response.
	Individualized intervention	Based on patient data, the platform should provide personalized exercise recommendations and interventions.
Professional support and collaboration	Medical team collaboration	The platform should support the cooperation between medical staff, so that doctors, nurses and rehabilitation therapists in different departments can participate in the postoperative rehabilitation management of patients.
	Educational resources sharing	The platform should provide rich educational resources, including sports rehabilitation knowledge, video tutorials, etc.
Platform function	User interface friendliness	Medical staff hope that the operation interface of the platform is simple and clear, easy to use, so that they can quickly view patient data and operate.
	Data security	Medical staff are very concerned about the security and privacy protection of patient data and meet the confidentiality requirements of medical data.

### 3.2 Main modules and functions of the remote exercise management platform

**Figure 2:**
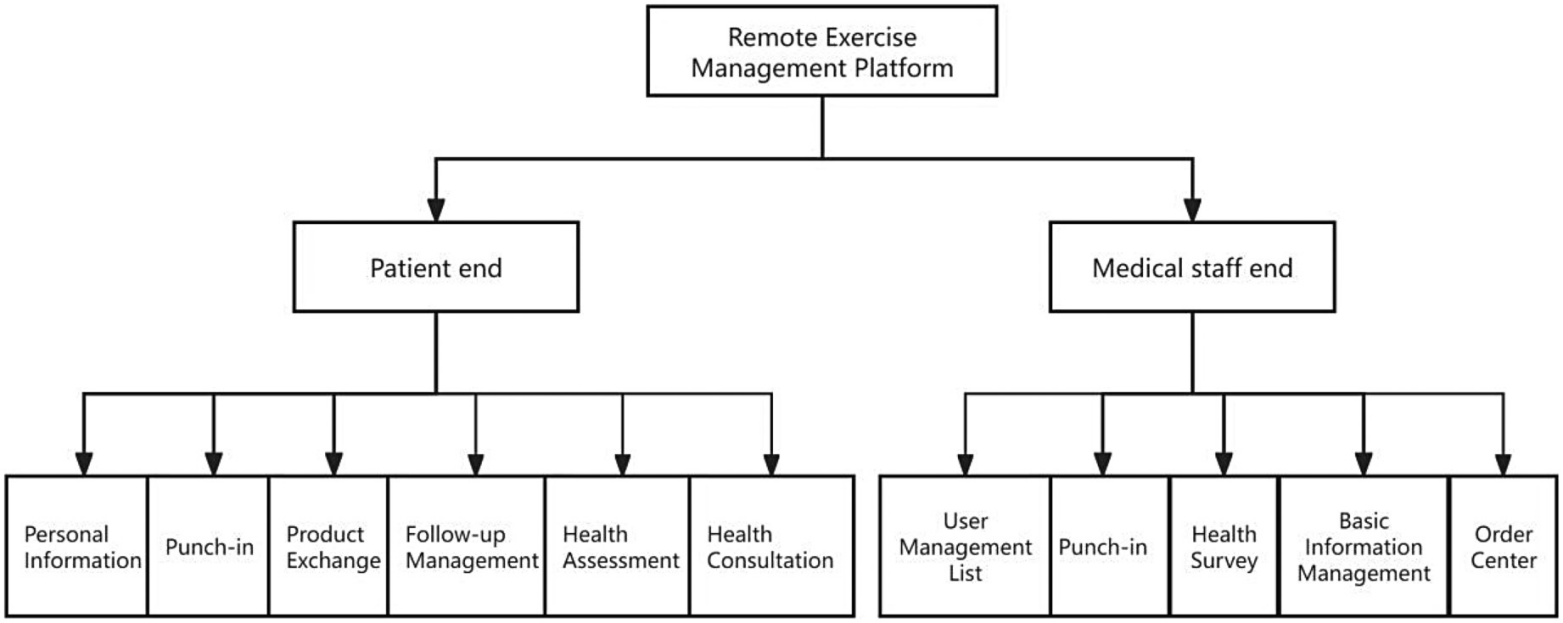
System architecture of remote exercise management platform.

The remote exercise management platform was divided into two main parts: the patient and medical ends (Figure 2). The user interfaces of the patients end is shown in Figure 2.

#### 3.2.1 Patient-end modules and functional descriptions

##### 3.2.1.1 Personal information

In the login interface, patients were required to fill in their basic personal information, which facilitated the implementation of targeted management of patients by the back-end medical staff. Information to be filled in includes name, gender, ID number (optional), age, contact information, hospitalization number, date of surgery, and type of disease.

##### 3.2.1.2 Rehabilitation punch-in

**Figure 3:**
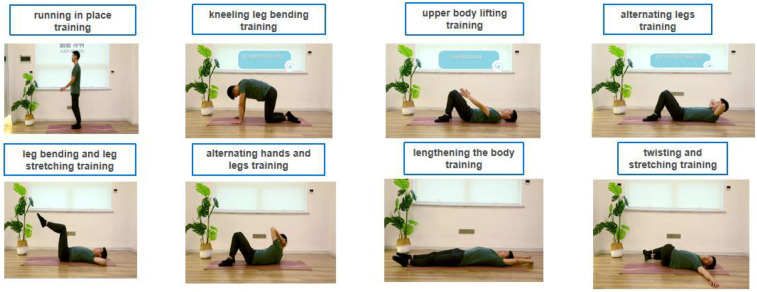
Exercise video schematic built-in the remote exercise management platform.

This module was the key module of this platform, which mainly involved the two functions of exercise learning and punch-in. The exercise learning module has 8 built-in videos (Figure 3). The content of the video was designed by a team of experts for the postoperative rehabilitation needs of colorectal cancer patients with intestinal stoma, with both authority and security, specifically including running in place training, kneeling leg bending training, upper body lifting training, alternating legs training, leg bending and leg stretching training, alternating hands and legs training, lengthening the body training, and twisting and stretching training. The exercise punch-in section required patients to record their follow-up videos and upload the recorded videos to the back-end to earn the corresponding exercise points.

##### 3.2.1.3 Product exchange

Patients can exchange their accumulated exercise points for corresponding intestinal stoma products in this module.

##### 3.2.1.4 Follow-up management

The follow-up management was conducted by medical staff. Their responsibilities included regularly reminding patients to engage in exercise rehabilitation, as well as inquiring whether patients encountered any difficulties or discomfort during their exercise routines.

##### 3.2.1.5 Health assessment

The module was regularly evaluated by medical staff using a professional assessment scale, which enabled them to fully understand the overall condition of patients and the specific effects of exercise rehabilitation.

##### 3.2.1.6 Health consultation

Patients could carry out consultations and messages related to disease management or sports precautions in this section, and the medical staff will answer questions in time after receiving the consultation content.

#### 3.2.2 Medical staff-end modules and functional descriptions

##### 3.2.2.1 User management list

Medical staff could view the patient's personal information in this module and improve or modify their personal information so that it can be maintained and updated in time.

##### 3.2.2.2 Rehabilitation punch-in

Medical staff could access and review the exercise videos uploaded by patients through this module. They assessed the standardization and safety of the patients' exercises. If it was determined that a patient's exercise technique required correction or if potential safety hazards were identified, the patient would be contacted to ensure the scientific validity and safety of their exercise regimen.

##### 3.2.2.3 Health survey

Medical staff could monitor whether patients regularly submit the relevant health assessment scales. If any abnormal results were detected, the patient or their caregiver would be promptly contacted for further verification and appropriate treatment.

##### 3.2.2.4 Order center

Medical staff could handle product exchange requests submitted by patients through this module and deliver the corresponding stoma products to the patient's home based on the address information provided.

### 3.3 Usability evaluation of the remote exercise management platform

#### 3.3.1 General information about patients in the usability evaluation

A total of 62 colorectal cancer patients with intestinal stomas were included in this usability evaluation. Among them, 23 (36.6%) were female, and 39 (63.4%) were male. There were 12 smokers (19.8%) and 48 non-smokers (77.4%). In terms of residential areas, 17 patients (27.6%) lived in urban areas, while 45 patients (72.4%) lived in rural areas. Specific results are presented in Table 4.

**Table 4:** General information about patients in the usability evaluation (*n* = 62).

Variable	Categorization	Cases	Component ratio
Age	60∼	24	38.9%
	70∼	20	48.6%
	80∼	8	12.5%
Gender	Female	23	36.6%
	Male	39	63.4%
Educational level	Junior high school and below	37	59.5%
	High school and above	25	40.5%
Smoking	No	50	80.2%
	Yes	12	19.8%
Drinking	No	48	77.4%
	Yes	14	22.6%
Tumor staging	Stage 1	14	23.3%
	Stage 2	20	32.3%
	Stage 3 and over	28	44.4%
Place of residence	Urban district	17	27.6%
	Rural district	45	72.4%
Type of work	Retirement	45	72.8%
	Employed	10	16.7%
	Jobless	7	10.5%
Marital status	Married	54	86.8%
	Divorce or single	8	13.2%

#### 3.3.2 Usability evaluation

The overall SUS score achieved was 88.39 ± 3.65. The mean scores for each of the ten items on the scale varied between 8.39 and 9.31. These detailed results are presented in Table 5. When compared to other mHealth tools, these results are quite promising. Typically, an SUS score above 68 is considered above average, suggesting that our system not only meets but exceeds the usability standards of many existing mHealth applications. The mean scores for each of the ten items on the scale, ranging from 8.39 to 9.31, further emphasize the robustness and reliability of our system across various usability dimensions. This comparison underscores the effectiveness of our design and implementation strategies in creating a user-friendly mHealth tool that is likely to be well-received by its target audience.

**Table 5:** The score of each item of the system availability scale.

Item	Mean	Standard deviation
1. I would like to use this small program often	9.31	1.29
2. I found this little program too complicated	8.87	1.33
3. I think this little program is easy to use	8.43	1.45
4. I think I need the help of technical staff to use this small program	9.15	1.19
5. I found that this small program integrates a variety of functions well	8.39	1.64
6. In the process of using the small program, I found that many operation results are inconsistent with the expected function	8.99	1.32
7. I think most users can quickly learn to use the applet	8.63	1.55
8. I found this little program is very awkward to use	8.83	1.34
9. I have great confidence in mastering this little program	8.67	1.48
10. Before using this small program, I need to learn a lot of knowledge	9.11	1.21

## 4 Discussion

### 4.1 Usability of the remote exercise management platform

The total SUS score in this study was 88.39, which was much higher than the overall average SUS score, representing a better usability of the remote exercise management platform. The design of platform, which integrates a comprehensive and systematic analysis of patient needs with literature reviews, patient interviews, medical staff interviews, and expert group discussions, has likely contributed to its high usability.

Further analysis of the SUS item scores revealed that the top two highest-scoring items were Item 1 and Item 4. In the SUS, the higher the score for odd-numbered items, the more the patient agrees with the viewpoint of the item, and the higher the score for even-numbered items, the more the patient disagrees with the viewpoint of the item.

Firstly, the the highest score was Item 1, which states “I would like to use this mini-program frequently.” As an odd-numbered item, this result indicates a high willingness among patients to use the platform. The reason may be that exercise management is a significant concern for colorectal cancer patients with stomas after discharge. The presence of a stoma changes their daily living habits, often leaving these patients in a dilemma between “wanting to exercise” and “being afraid to exercise.” Therefore, patients have a strong desire to obtain professional advice and guidance on exercise from healthcare providers (23). The development of the remote management platform effectively addresses the exercise management needs of colorectal cancer patients with intestinal stomas after discharge. This alignment likely contributes to the high score for Item 1.

Item 4 (“I think I need technical support to use this mini-program”) achieved the second-highest score, with patients strongly disagreeing with this statement. Since Item 4 is an even-numbered item on the SUS, this high score indicates exceptional perceived ease of use and minimal need for technical assistance. This notable usability likely stems from our rigorous patient-centered design approach. The interface architecture was specifically engineered to minimize cognitive burden through elimination of unnecessary navigational steps and operational complexities. Module nomenclature incorporated straightforward, accessible terminology to facilitate immediate comprehension and reduce potential confusion among users with varying levels of technological proficiency. Furthermore, acknowledging the demographic composition of colorectal cancer patients, which frequently includes older adults with potentially limited digital literacy, the platform incorporates a collaborative account management system enabling authorized caregivers to assist with platform navigation and data entry when needed. This feature effectively addresses potential age-related technological barriers while preserving patient autonomy in rehabilitation management. Collectively, these human-centered design elements substantially enhance the platform's clinical utility and accessibility across diverse patient populations, contributing to its exceptional usability metrics.

### 4.2 Strengths and limitations of the remote exercise management platform

The remote exercise management platform offers several distinct advantages. Primarily, it provides a comprehensive yet focused intervention specifically designed for colorectal cancer patients with intestinal stomas during their post-discharge rehabilitation phase. The platform's core functionality facilitates regular exercise regimens that promote disease recovery while effectively preventing stoma-related complications. Complementary functions, including health consultation, systematic assessment, and structured disease follow-up, ensure patients maintain access to high-quality healthcare resources following hospital discharge. Previous studies have also reached conclusions consistent with those of this study, suggesting that telehealth platforms can assist patients in better disease recovery. Onyeaka et al. found that cancer survivors who had health applications installed on their mobile device were more likely to meet national recommendations for diet (fruit and vegetable consumption) and strength training than those without health apps (24).

A key strength of the platform lies in its evidence-based, multidisciplinary approach to content development. The eight exercise tutorial videos were meticulously designed through collaborative effort among rehabilitation medicine specialists, colorectal surgeons, certified enterostomal therapists, sports medicine physicians, and yoga physiotherapists. This integration of diverse clinical expertise ensures the exercises address the unique rehabilitation needs of patients with colostomies. Furthermore, the platform's requirement for patients to upload recorded exercise sessions enhances intervention adherence while enabling remote assessment of exercise quality and safety, thereby maximizing therapeutic efficacy. The findings from previous studies further support the conclusions of this study. A systematic review of Su et al. concluded that Home-based exercise interventions are feasible and safe for people diagnosed with cancer (25). Ayyoubzadeh et al. pointed that sensor-based telemonitoring systems for patients with colorectal cancer after surgery are possible solutions that can make the process automatic for patients and caregivers. The apps for remote colorectal patient monitoring can be designed to be useful (26).

Despite these strengths, several limitations warrant consideration for future platform refinement. Most notably, current medical resource constraints preclude real-time patient-provider interaction. Future iterations should optimize resource allocation and integrate artificial intelligence technologies to facilitate synchronous communication between healthcare providers and patients, enhancing the platform's responsiveness to emerging patient needs.

Additionally, the current health assessment module relies predominantly on validated subjective measures rather than incorporating objective physiological parameters. Future development should integrate commercially available wearable technologies such as accelerometers and biometric monitoring devices to collect objective behavioral and physiological data. This integration would enable more precise activity quantification, facilitate personalized exercise prescription, and potentially improve adherence through data-driven feedback mechanisms. Such technological enhancement would significantly advance the platform's capacity to deliver tailored rehabilitation guidance for this vulnerable patient population (27, 28).

It is important to acknowledge the limitations inherent in this study. First, the varying levels of digital literacy among patients could significantly impact the results. Patients with lower digital skills may not utilize the health apps as effectively, potentially affecting the study's outcomes. Second, the study's conclusions might not be universally applicable. The specific context and demographic characteristics of the participants may limit the generalizability of the findings to other settings or populations. Last, the SUS is a reliable tool for measuring perceived usability, the clinical effectiveness of the platform remains to be established.

## 5 Conclusion

In response to the rehabilitation needs of patients with intestinal stomas following colorectal cancer surgery, we developed a comprehensive remote management platform specifically designed to promote exercise in this population. The platform incorporates multifaceted functionality, evidence-based content with rigorous medical validation, and demonstrates high usability based on systematic evaluation. Future iterations could integrate artificial intelligence technologies to enhance real-time patient-provider interactions. Additionally, incorporating commercially available wearable devices into the platform's health assessment module would enable precise monitoring of patients' physiological parameters and activity metrics, facilitating the delivery of more personalized, data-driven health guidance. This technological approach represents a promising strategy to address the unique rehabilitation challenges faced by colorectal cancer survivors managing intestinal stomas.

## Data Availability

The raw data supporting the conclusions of this article will be made available by the authors, without undue reservation.

## Appendix 1 The items in System Usability Scale (SUS)

1. I think that I would like to use this system frequently.

2. I found the system unnecessarily complex.

3. I thought the system was easy to use.

4. I think that I would need the support of a technical person to be able to use this system.

5. found the various functions in this system were well integrated.

6. I thought there was too much inconsistency in this system.

7. I would imagine that most people would learn to use this system very quickly.

8. I found the system very cumbersome to use.

9. I felt very confident using the system.

10. I needed to learn a lot of things before I could get going with this system.
